# The Effect of Hydraulic Loading Rate and Influent Source on the Binding Capacity of Phosphorus Filters

**DOI:** 10.1371/journal.pone.0069017

**Published:** 2013-08-02

**Authors:** Inga Herrmann, Amir Jourak, Annelie Hedström, T. Staffan Lundström, Maria Viklander

**Affiliations:** 1 Department of Civil, Environmental and Natural Resources Engineering, Luleå University of Technology, Luleå, Sweden; 2 Division of Fluid and Experimental Mechanics, Luleå University of Technology, Luleå, Sweden; Dowling College, United States of America

## Abstract

Sorption by active filter media can be a convenient option for phosphorus (P) removal and recovery from wastewater for on-site treatment systems. There is a need for a robust laboratory method for the investigation of filter materials to enable a reliable estimation of their longevity. The objectives of this study were to (1) investigate and (2) quantify the effect of hydraulic loading rate and influent source (secondary wastewater and synthetic phosphate solution) on P binding capacity determined in laboratory column tests and (3) to study how much time is needed for the P to react with the filter material (reaction time). To study the effects of these factors, a 2^2^ factorial experiment with 11 filter columns was performed. The reaction time was studied in a batch experiment. Both factors significantly (α = 0.05) affected the P binding capacity negatively, but the interaction of the two factors was not significant. Increasing the loading rate from 100 to 1200 L m^−2^ d^−1^ decreased P binding capacity from 1.152 to 0.070 g kg^−1^ for wastewater filters and from 1.382 to 0.300 g kg^−1^ for phosphate solution filters. At a loading rate of 100 L m^−2^ d^−1^, the average P binding capacity of wastewater filters was 1.152 g kg^−1^ as opposed to 1.382 g kg^−1^ for phosphate solution filters. Therefore, influent source or hydraulic loading rate should be carefully controlled in the laboratory. When phosphate solution and wastewater were used, the reaction times for the filters to remove P were determined to be 5 and 15 minutes, respectively, suggesting that a short residence time is required. However, breakthrough in this study occurred unexpectedly quickly, implying that more time is needed for the P that has reacted to be physically retained in the filter.

## Introduction

It has been suggested that filters for the removal and recovery of phosphorus (P) from wastewater would be beneficial for on-site wastewater treatment e.g. in constructed wetlands. Many filter materials have been investigated [1], [2] but it is still uncertain how well they would work in full-scale installations. Long-term field studies are not widely available [1]. Usually, P binding capacity is determined from laboratory batch or fixed-bed column experiments and used to calculate the filter’s lifetime. To speed up the laboratory testing, parameters such as influent source (real wastewater or synthetic P solution), hydraulic loading rate, residence time, P loading and temperature do not match field conditions. These parameters vary substantially between different studies although it has been shown that the method used to test the materials influences the measured P binding capacity and thus the predicted performance of the filters as well as their predicted lifetime [3]. There is a need for a robust test method that can be used to investigate filter materials and that gives reliable estimates of how well and how long they will function under given conditions. Therefore, it needs to be investigated as to which parameters can be altered in the laboratory to determine P binding capacity in a reasonable amount of time whilst assuring that the binding capacity determined in the laboratory resembles the one expected under field conditions.

Hydraulic loading rate and hydraulic residence time are closely connected and it has been shown that there is a relationship between these parameters and P binding capacity [1]. The effect of varying residence time has been discussed by Brooks, Rozenwald et al. (2000) who stated that residence times from 15 to 143 hours (corresponding to loading rates of 905 and 114 L m^−2^d^−1^) positively affect the percentage of P removed from wastewater by wollastonite. Ádám et al. [4] suggested that the hydraulic loading rate does not affect the P binding capacity in *Filtralite P*, however, increased loading rate led to a faster breakthrough of P in their study. Also, the hydraulic loading rate was found to increase P binding capacity in *Filtra P* (unpublished data). The results of these studies show that the effect of hydraulic loading rate has not been comprehensively investigated. In particular, it is still unclear if loading rate or residence time is responsible for variations in binding capacity.

The measured P binding capacity can also vary depending on whether wastewater or synthetic P solution is used in the test: using wastewater decreases P binding. This effect has been shown in batch experiments with blast furnace slag [5] and a column experiment with *Filtralite P* [6]. However, the conditions in which the batch experiments performed by Hedström and Rastas [5] were carried out did not resemble field conditions at all and the experiment by Ádám et al. [6] was performed with only two filter columns that were fed with different influent P concentrations which could have confounded the effect of the influent source. Therefore, the effect of influent source on P binding capacity still needs to be evidenced. Additionally, the effect has not yet been quantified.

The present study contributes to developing a sound testing method by investigating the effect of influent source and hydraulic loading rate on the determined P binding capacity. The objectives of this study are to investigate and quantify the effects of hydraulic loading rate and influent source (wastewater and phosphate solution) on P binding capacity and to study how much time is needed for the P to react with the filter material.

## Materials and Methods

Two laboratory experiments were performed: a batch experiment to determine the time needed for the P to react within the filter material and a factorial column experiment to investigate the effect of different influent sources and hydraulic loading rates on the P binding capacity of the material.

### 1. Ethics statement

Oral permission for sampling wastewater at Sundom’s wastewater treatment plant was issued by Luleå municipality, Technical Department.

### 2. Filter material

The filter material used for the experiments was Filtralite P^®^. Its elemental composition was determined using inductively coupled plasma (ICP) analysis (Table 1). According to the product data sheet, Filtralite P^®^ has a porosity of ca. 0.6 and a bulk density of 370 kg m^−3^.

**Table 1:** Elemental composition of Filtralite P^®^.

Element	Content [g kg^−1^]
Si	269
Al	86.1
Fe	56.5
Ca	35.7
K	29.2
Mg	28.3
S	0.24

### 3. Experiment to determine the time needed for the P to react

The time needed for the P to react with the filter material was determined with two series of batch experiments performed in dublicate (n = 2): one was conducted using phosphate solution and the other one using wastewater. The concentration of dissolved P was 12 mg L^−1^ in the phosphate solution and 11 mg L^−1^ in the wastewater (the wastewater used is further described in 2.4.3). The conditions in this experiment should resemble those in full-scale filters in terms of the liquid to solid ratio. This was 1.77±0.05 L kg^−1^ and was made by weighing 201.9 g of material (corresponding to ca. 200 g of dry material) into 1L-glass beakers. Then, 353.5±9.0 g of phosphate solution was added to one set of beakers and 356.7±7.9 g of wastewater was added to the other set of beakers until the material was almost covered but did not start floating up. Beakers were covered with plastic sheets and left standing at room temperature for 5 min, 15 min, 30 min, 1 h, 2 h, 4 h, 8 h and 16 h. The liquid phase was then poured through a 0.5 mm sieve, analyzed with respect to pH and redox potential, further filtered through a 0.45 μm filter and analyzed with respect to dissolved P.

### 4. Factorial column experiment

#### 4.1. Factorial design

The effect of influent source and hydraulic loading rate on the P binding capacity of the filter material was investigated in a laboratory experiment with a 2^2^ full factorial design [7] with replicates (n = 2) and 3 centre points (one with wastewater and two with phosphate solution, Table 2). In total, 11 filters were tested (Table 2). The influents used were urine-spiked wastewater and synthetic phosphate solution. The low, medium and high levels of hydraulic loading rates and the corresponding flows, residence times and filter velocities are shown in Table 2.

**Table 2:** Filter columns and their factor settings.

	Factor					
	Loading rate		Influent source	Flow	Residence time^a^	Filter velocity
Filter number	Level	L m^−2^ d^−1^		[L d^−1^]	[min]	[cm d^−1^]
A1, A2	Low	94±3	Wastewater	0.41±0.01	575±16	9±0.3
B	Medium	612	Wastewater	2.63	89	61
C1, C2	High	1056±33	Wastewater	4.54±0.14	51±2	106±3
D1, D2	Low	97±3	P solution	0.42±0.01	560±15	10±0.3
E1, E2	Medium	651±21	P solution	2.80±0.09	83±3	65±2
F1, F2	High	1188±46	P solution	5.11±0.20	46±2	119±5

a residence time  =  pore volume divided by flow.

#### 4.2. Experimental set-up

11 acrylic glass columns with a diameter of 7.4 cm were used in the experiments and run in up-flow mode using peristaltic pumps. For even flow distribution, a ca. 3 cm thick layer of glass beads (diameter 4 mm) was placed at the bottom of the columns. 100 g of oven-dried (105°C) and desiccator-cooled filter material were placed on top of these. A funnel filled with glass beads was put on top of the columns to ensure a smooth outflow. The effluent was collected in plastic containers.

#### 4.3. Influent solution

The ideal P concentration of the phosphate solution and secondary wastewater used in the experiments was 12 mg L^−1^; this resembles the P concentrations of secondary effluent to be treated by on-site P filters that can be between *ca.* 5 and 14 mg L^−1^ [8]. The phosphate solution was prepared by dissolving KH_2_PO_4_ in distilled water.

Secondary wastewater was sampled twice weekly from a nearby wastewater treatment plant serving 300 pe. At the plant, the wastewater had been subjected to screening, primary sedimentation and biological treatment in a tower trickling filter. As the P concentration in the wastewater was below the target concentration, it was increased by adding human urine. The total and dissolved P concentrations in the urine-spiked wastewater were 13±3.6 and 11±1.7 mg P L^−1^, respectively. The pH was 7.1±0.2, the redox potential 127±31 mV, the TSS content 87±104 mg L^−1^ and the turbidity 56±66 NTU. The contents of total organic carbon (TOC) and dissolved organic carbon (DOC) were 126±34 mg L^−1^ and 108±8 mg L^−1^, respectively. The high deviations in TSS and turbidity in the wastewater were due to a temporarily non-functioning sludge pump in the pre-sedimentation at the treatment plant. The phosphate solution had an average concentration of 13±0.6 mg phosphate-P L^−1^, a pH of 5±0.4 and a redox potential of 304±48 mV. The above stated means and standard deviations were calculated over the entire experimental run-time although some filters were saturated earlier.

#### 4.4. Effluent sampling

Effluent samples were taken at least twice weekly from the plastic containers for each column. High and medium flow columns were sampled more frequently at the beginning. Samples were analyzed with respect to total and dissolved (filtered through a 0.45 μm filter) P, turbidity and TSS. Total and dissolved (filtered through a 0.45 μm filter) organic carbon (TOC and DOC) were only analyzed in samples from the wastewater-fed filters. pH and redox potential were measured from grab samples of the outflow taken at the same time as the effluent samples.

#### 4.5. Evaluation

A P filter can generally be described as well-functioning as long as the outflow P concentrations are below 1 mg L^−1^, which is the normal limit used for small systems in Norway [9]. Therefore, breakthrough of P through the filters was defined as to have happened when the concentration of dissolved outflow P divided by the concentration of dissolved inflow P was ≥0.08. The filters were assumed to be saturated when the dissolved P concentration in the outflow divided by the dissolved P concentration of the influent was ≥0.92. Moving averages were used to determine the point of saturation. The P_tot_ binding capacity was calculated using the total P concentrations of the influent and effluent:

P_tot_ binding capacity 
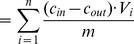
 (1)

where

P_tot_  =  binding capacity in g kg^−1^

n  =  number of samples taken

c_in, out_  =  total P concentration of the influent and effluent [mg L^−1^]

V_i_  =  volume of wastewater or P solution that has passed through the filter between sample i-1 and i [L]

m  =  mass of the filter material [g].

Data were evaluated using analysis of variance and multiple linear regression with the MODDE software package [10]. The significance level was set to α = 0.05.

### 5. Analyses

The pH was measured using a WTW pH330 pH meter with a WTW SenTix41 pH electrode. The redox potential was measured using a pHM95 pH/ion meter (Radiometer, Copenhagen). TSS was determined following the European standard EN 872: 2005 [11]. Turbidity was measured using an HACH 2100N turbidimeter (nephelometric method). All P analyses were performed using a Quattro spectrometer and the device-specific method no. A-031-04, following the European standards (ascorbic acid method) with modified digestion (persulfate oxidation) [12]. Organic carbon was analysed using a Hach Lange photometric TOC analyzer.

## Results

### Time needed for P to react

**Figure 1:**
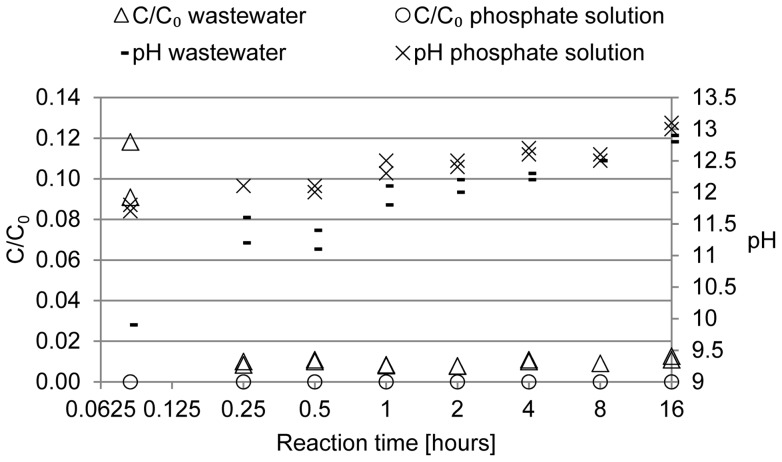
Results of the experiment to determine the time needed for the P to react. Ratio of dissolved P concentration and initial dissolved P concentration (C/C_0_), and pH in the wastewater and phosphate solution versus contact time.

Using phosphate solution, the concentration of dissolved P could be decreased to below detection limit in the solution in less than 5 minutes (Fig. 1). The P detection limit was 0.05 mg L^−1^. When wastewater was used, it took longer to remove the phosphate from solution and the concentration could not be decreased that much: after 5 min, 10±2% of the P remained in solution and it took 15 minutes to remove about 99% of it (Fig. 1). The pH was generally lower when wastewater was used with the difference in pH being greater for shorter contact times (Fig. 1). The redox potential decreased logarithmically with contact time, from 11±11 mV (after 5 min) to −84±6 mV (after 16 h) in the wastewater and from 29±6 mV (after 5 min) to −74±11 mV (after 16 h) in the phosphate solution.

### Factorial column experiment

**Figure 2:**
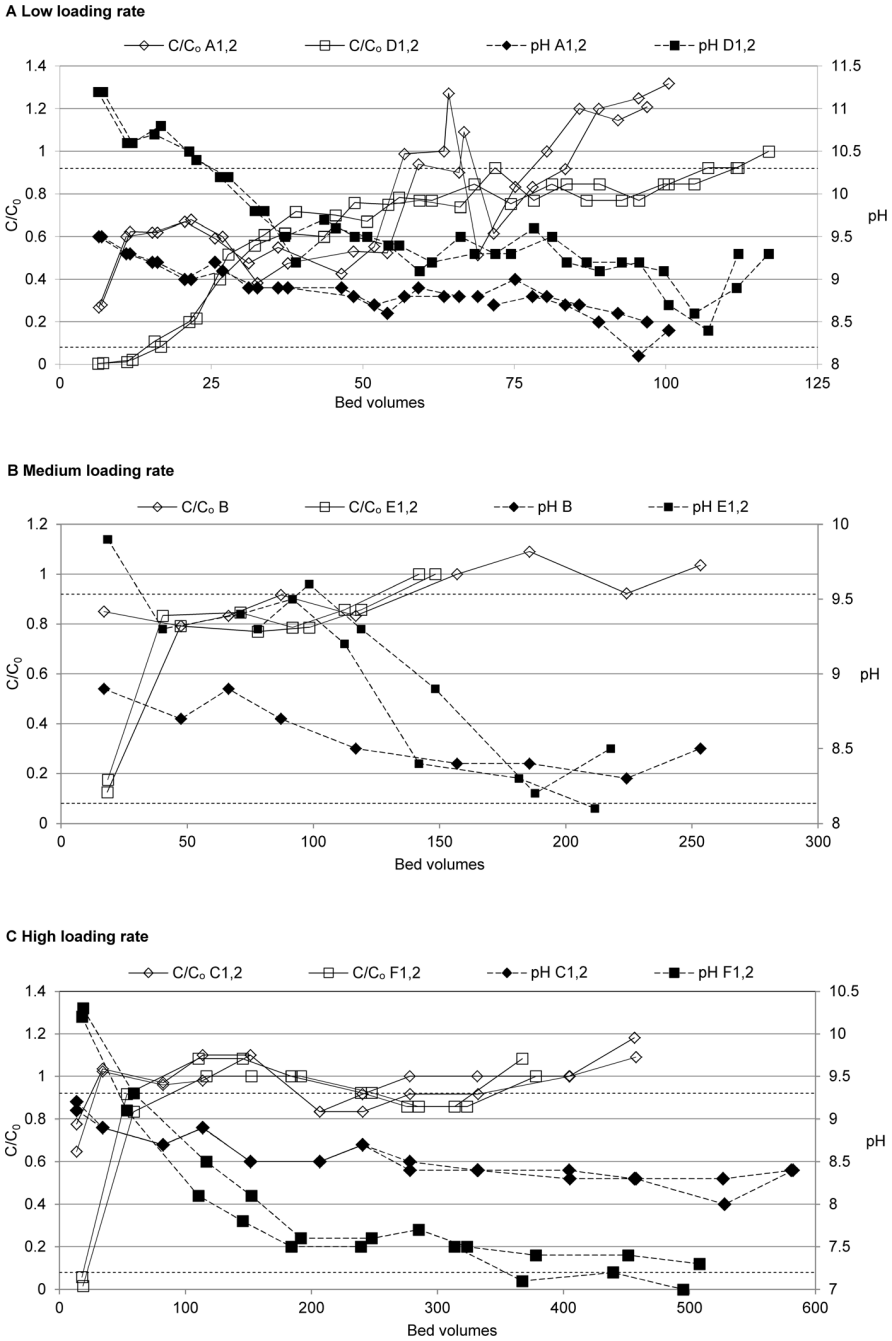
P breakthrough curves and pH in the effluent of the column experiment. Ratios between effluent and influent dissolved P concentration (unfilled markers) and pH (filled markers) over bed volumes for low loading rate filters (A), medium loading rate filters (B) and high loading rate filters (C). The horizontal dashed lines show the criteria for breakthrough and saturation.

Dissolved P effluent concentrations and pH for the three loading rates are shown in Fig. 2. Dissolved P concentrations in the effluent from the wastewater filters were generally higher when compared to the phosphate solution filters. Already at the first sampling occasion, these concentrations were, for wastewater (P solution) filters, 2.3 (0.06), 8.5 (1.8) and 6.1 (0.5) mg L^−1^ for low, medium and high loading rates, respectively (Fig. 2). Breakthrough was only observed for filters D1, 2 and F1, 2 (Fig. 2). In the other filters, breakthrough occurred before the first sample was taken. The pH at breakthrough for D1 and D2 was 10.8 and 10.7; for F1, 2 it was 10.2 (Fig. 2).

**Figure 3:**
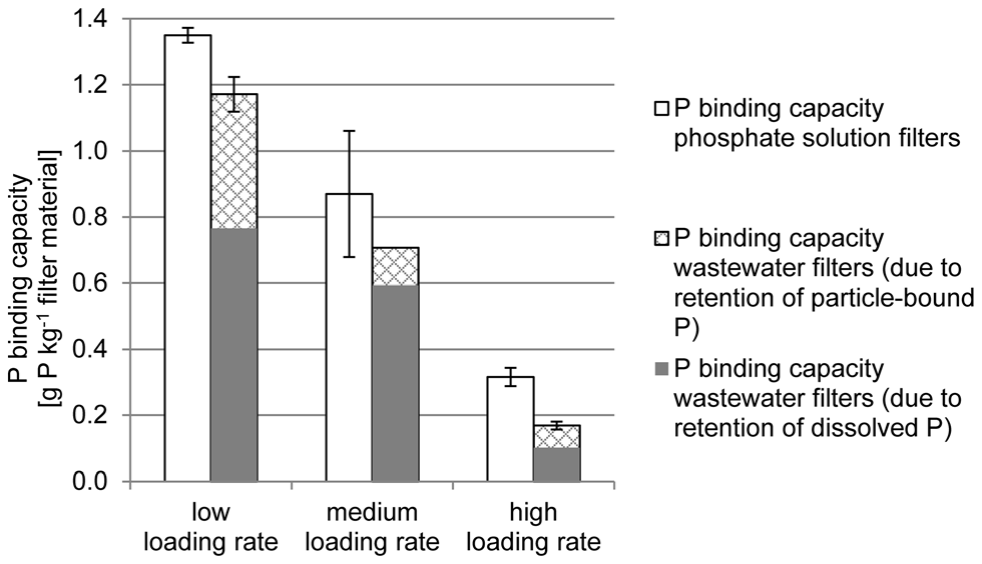
P_tot_ binding capacity for phosphate solution and wastewater filters at the three loading rates applied. The P binding capacity of the wastewater filters is made up of two parts: one part is due to the uptake of dissolved P (gray bars) and the other part is due to the uptake of particle-bound P (cross-hatched bars).

**Figure 4:**
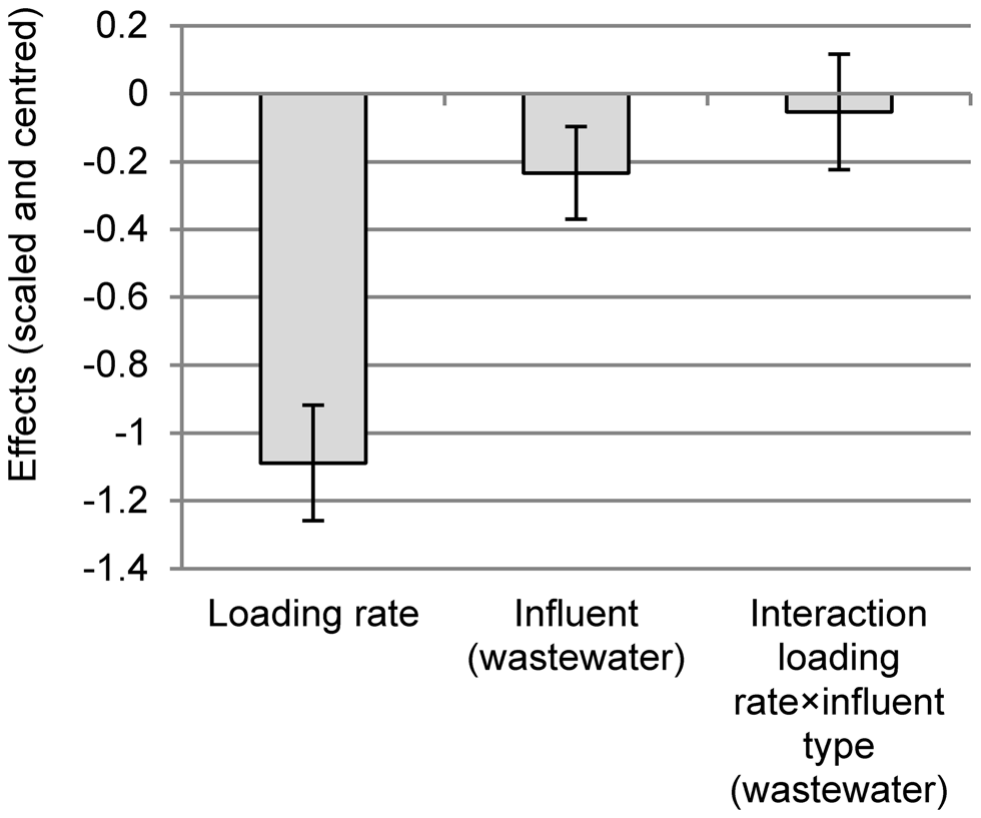
Scaled and centred effects of the investigated factors on P_tot_ binding capacity. The error bars show the 95% confidence interval on the effect value. The scaling of the data makes the coefficients comparable.

The P binding capacities determined for the filters are shown in Fig. 3. A small part of the P binding capacity in wastewater filters was due to the retention of particle-bound P. Both hydraulic loading rate and influent source had a significant negative effect on the P_tot_ binding capacity whilst the interaction effect of these two factors was not significant (Fig. 4). The effect of loading rate was stronger than the effect of influent source.

A linear model based on the factor main effects was fitted to the P_tot_ binding capacity data (Eq. 2). The fit was good (R^2^_adjusted_  = 0.96) which means there is a strong linear relationship between the two factors and P_tot_ binding capacity. In Eq. 2, *influent source* must be set to −1 for wastewater filters and to +1 for P solution filters; the unit of loading rate is L m^−2^ d^−1^. The equation can only be used within the levels of loading rate (and the corresponding residence times) investigated in this study.
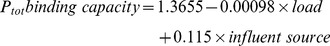
(2)


Eq. 2 can be used to demonstrate the effects of influent source and loading rate by quantification: when increasing the loading rate from 100 to 1200 L m^−2^ d^−1^, P_tot_ binding capacity decreased from 1.152 to 0.070 g kg^−1^ for wastewater filters and from 1.382 to 0.300 g kg^−1^ for phosphate solution filters (Eq. 2). Holding the loading rate at the low level, the average P_tot_ binding capacity of wastewater-fed filters was 1.152 g kg^−1^ as opposed to 1.382 g kg^−1^ for phosphate solution filters. As the model fits the data well, these calculated values are very close to the P_tot_ binding capacities shown in Fig. 3.

pH generally decreased over time in all filters (Fig. 2, Table 3). Wastewater filters had a significantly lower start pH and mean pH compared to phosphate solution filters. When the filters were saturated, the pH was equally low for all filters (on average 8.7±0.3, Table 3). The redox potential was positive for all filters throughout the experiment; the average redox potential was significantly lower in the wastewater filters compared to phosphate solution filters. For phosphate solution filters, increasing the loading rate led to an increase in mean redox potential which was not observed for the wastewater filters (Table 3).

**Table 3:** Effluent parameters for the filters and influent concentration of particulate P.

		Wastewater filters			P solution filters		
Parameter		A1,2	B	C1,2	D1,2	E1,2	F1,2
Loading rate		low	med	high	low	med	high
Number of treated BV until saturation	Mean	65	117	85	112	145	113
	SD	2	–	34	0	5	4
Start pH	Mean	9.5	8.9	9.2	11.2	9.9	10.3
	SD	0.0	–	0.1	0.0	0.0	0.1
Average pH	Mean	9.0	8.7	9.0	9.6	9.3	9.3
	SD	0.0	–	0.0	0.0	0.1	0.2
End pH	Mean	8.8	8.5	8.8	9.1	8.7	8.5
	SD	0.0	–	0.1	0.3	0.4	0.0
Average redox potential [mV]	Mean	85	90	82	108	127	183
	SD	2	–	7	9	6	5
Average TSS [mg L^–1^]	Mean	8.1	14.1	13.6	1.0	2.3	3.2
	SD	1.3	–	5.1	0.7	0.3	0.0
Average turbidity [NTU]	Mean	4.6	7.1	7.0	0.7	3.1	8.3
	SD	1.1	–	1.2	0.0	0.3	1.2
Average concentration of particulate P [mg L^–1^]	Mean	0.6	0.9	0.6	0.2	0.6	1.1
	SD	0.1	–	0.8	0.1	0.0	0.6
Influent concentration of particulate P [mg L^–1^]	Mean	2.9	1.2	1.3	–	–	–
	SD	0.0	–	0.2	–	–	–

Filters shown in one column are replicates (SD: standard deviation of replicates, BV: bed volumes).

Particle-bound P escaped from the filters, which is shown by the concentration measurements of particle-bound P as well as TSS and turbidity measurements in the effluent (Table 3). For all but the low loaded phosphate solution filters, turbidity and TSS were significantly positively correlated. The turbidity and the total mass of TSS in the effluent were significantly higher in wastewater filters, and turbidity significantly increased with increasing loading rate. Both TSS and the average concentration of particle-bound P in the effluent showed an increasing trend over loading rate (Table 3).

The reduction of TOC and DOC in the wastewater filters was 71% and 75%, respectively. One-way analyses of variance showed no significant effect of loading rate on the effluent TOC, DOC and particulate OC nor on the reduction of TOC and DOC (in %) in the filters.

## Discussion

### Reliability of produced data

In this study, a replicated factorial design was used producing a solid and valid evaluation. The effect of influent source and loading rate could be quantified using a regression model (Eq. 2) and also tested for statistical significance. The regression was significant (p = 0.000) and the high value of R^2^_adjusted_  = 0.96 indicates a good fit to the data. The model is valid (showed no lack of fit). The replicated filters generally showed very similar responses (Fig. 2, Fig. 3, Table 3). The pure error was therefore very small indicating a high reproducibility of the experiment. For the aforementioned reasons, the estimations of the factor effects made in this study can be considered reliable.

When the effect of different factors is to be investigated, a solid design is necessary to enable a comparison between the levels of those factors. The factors studied here, influent source and loading rate, have been previously studied, although their effects were not the main focus of those studies. Therefore, the comparison between their levels has been difficult either because of the lack of replicates and too few observations at each level [6], [13], or because standard deviations were not presented [4], or because the effects were influenced by nuisance factors such as different influent P concentrations [6].

### Effect of loading rate

The loading rate affected the determined P_tot_ binding capacity in this study negatively (Fig. 4). To understand the negative effect of loading rate in this study, it is important to look at the processes in the filter. When the filter material makes contact with water, calcium (Ca) ions probably hydrolyze through the weathering of Ca-containing compounds in the material, reacting with phosphate and precipitate. The precipitates are then physically/mechanically retained in the filter. The negative effect of loading rate could be explained by an increased wash-out of P precipitates. In this study, the concentration of particle-bound P in the effluent showed an increasing trend with loading rate (Table 3) which means that more particle-bound P was washed out at higher loading rates. This effect was observed for both influent sources, however it was less pronounced for the wastewater filters because of the retention of particles contained in the influent wastewater. Influent particles in the wastewater that accumulated in the filter might also have enhanced the filters’ ability to filtrate (small) particles. Another explanation for the lower P_tot_ binding capacity at high loading rates could be the washout of reactive Ca ions which has been observed in a study by Ádám et al. [4]. The negative effect of loading rate could also be explained by a poor contact between influent and filter particles: Suliman et al. [14] showed with tracer tests that increasing the loading rate can lead to a higher preferential flow and less porosity used for active flow.

Increasing the loading rate also implies a shorter residence time of the solution in the filters. Filtralite P^®^-filters are usually constructed as subsurface horizontal flow filter beds implying that the area used for the calculation of the hydraulic loading rate would be the cross-sectional area. It can be debated as to whether residence time or loading rate is the relevant design parameter for filters. Most probably, both parameters are of importance: loading rate is the factor governing the pore velocity in the filter and can thus influence surface reactions on the material as well as the fixation of Ca-P precipitates on the surfaces of the filter particles and between particles. Residence time, on the other hand, determines the time available for Ca-P precipitates to form and to form bigger particles e.g. hydroxyapatite (Hap) crystals. This study shows that the time needed for the formation of Ca-P compounds is short (Fig. 1). Also, Hap has been shown to form within minutes at pH 9 in the presence of boehmite [15]. This indicates that a short residence would be sufficient and the hydraulic loading rate would be the more relevant parameter. However, P breakthrough in the filters occurred quickly: before the first samples were taken for all wastewater filters and for filters E1, 2 (Fig. 2) although the first samples were taken after 5 days for the low loading rate filters, after 2 days for the medium loading rate filters and after 1 day for the high loading rate filters. This rapid breakthrough was unexpected because the levels of loading rate chosen (96, 638 and 1122 L m^−2^ d^−1^) were well in line with the hydraulic loading rates used in full-scale Filtralite P^®^-facilities that used loading rates of 90 to 160 L m^−2^ d^−1^ [9], 222 L m^−2^ d^−1^ [16], 250 L m^−2^ d^−1^ [17] and 444 L m^−2^ d^−1^ [18]. Very high loading rates of several thousands of L m^−2^ d^−1^ have been applied in some facilities that have removed 99% P in the first three years [19]. Therefore, the loading rates used in this study seem justified. However, the size of the filter bed we used was small (100 g of material per filter column) and therefore, the residence times of the filters were short (49 min, 85 min and 9.5 h). The previously mentioned full-scale facilities use residence times of about 1.7 days [19], 2.9 to 5.6 days [9] and about 18 days [16]. Therefore, despite the short reaction time (Fig. 1), the residence time seems to play a major role in P retention and might, for the material investigated, be the more relevant parameter. Possibly, the residence times connected to the chosen loading rates in this study were too short for the precipitates to agglomerate or crystallize to particles that were big enough to be physically retained in the filter matrix. Also, mechanisms other than Ca-phosphate precipitation might contribute to the retention of P in the material. In used Filtralite^®^ P, Ádám et al. [16] found P bound to surfaces of aluminium-containing particles. These surface processes might take more time and therefore require a longer residence time.

### Effect of influent source

Although the effect of the different influent sources was less pronounced than the effect of loading rate, the P_tot_ binding capacity in the wastewater filters was significantly lower compared to phosphate solution filters (Fig. 4). This agrees with the findings of Ádám et al. [6] whose P solution-fed column treated a higher cumulative amount of P before breakthrough compared to their wastewater-fed column. Three possible explanations for this will now be discussed: a different pH regime, inhibition of Ca-phosphate formation by humic substances and clogging of the filter by accumulation of particles with the formation of a biofilm.

Both the start pH and average pH (over the experimental runtime) were significantly lower in the wastewater filters (Fig. 2, Table 3) which could explain the higher outflow P concentrations in the wastewater filters right from the beginning and thus the early breakthrough and the lower P_tot_ binding capacity. A great part of the soluble organics in wastewater are volatile acids, most of them being acetic acids [20]. The wastewater is a buffer solution in itself, thus decreasing the buffer effect of the material resulting in a lower outflow pH compared to the phosphate solution filters. Ca-phosphate precipitation, however, is favoured at high pH [21]. Song et al. [22] found that the saturation index of Hap is a polynominal function of the solution pH and increases when pH increases from 7 to 11. Hap is a stable Ca-phosphate phase that is likely to be formed in P filters and has been found in filter materials after use [23], [24].

The average pH in the wastewater filters was 8.7 and 9.0 (Table 3), which is the pH range within which humic substances can inhibit the precipitation of Ca-phosphates. The inhibitory effect is pronounced at pH 8, small at pH 9 and non-existent at pH 10 [23]. This effect has been attributed to the combination of humic substances with Ca, but also to the blocking of active growth sites on newly nucleated Ca-phosphate precipitates by organic matter [23].

The reduction of DOC in the wastewater filters was as high as 75% (see chapter 3.2) indicating a sorption of DOC in the filters. It has previously been suggested that the formation of Ca-phosphate precipitates is responsible for the removal of dissolved organic matter: newly formed Ca-phosphate precipitates have a large surface area and adsorb the organic matter [23]. Particulate organic matter was removed by filtration, indicated by an average TOC reduction of 71%. The retained organic matter in the filters may have clogged the filter and contributed to the formation of a biofilm layer on the filter particles which possibly hampered the dissolution of Ca from the material. If less Ca is dissolved, the Ca/P ratio is lower. Song et al. [22] found the Ca/P ratio to increase the saturation index for Hap. As Hap is an important reaction product, the Ca/P ratio may be a relevant parameter governing P removal. Song et al. [23] further suggested that organic matter (other than humic substances) consumes Ca but is not precipitated, which would mean in this case that Ca may have been washed out by organic matter, thus decreasing the Ca/P ratio further. Biofilm formation and decreased Ca dissolution could therefore explain the lower P binding capacity (Fig. 3) and lower numbers of bed volumes that could be treated with the wastewater filters (Table 3).

### Factor settings in laboratory filter tests

In one way or another, laboratory tests need to be accelerated in comparison to full-scale filters to obtain results in a reasonable amount of time. Therefore, at least one filter parameter must change. Hydraulic loading rate seems to be an unsuitable parameter to adjust in order to decrease the time needed for testing, as it had a significant negative effect on the P_tot_ binding capacity in this study (Fig. 4) and previous studies [4], [13]. Increasing the influent P concentration can affect P binding capacity both negatively (unpublished data) and positively [4], although in the former study data were evaluated until breakthrough whilst in the latter, data until saturation were used. Using a synthetic P solution, instead of wastewater, would overestimate the determined P binding capacity because of the negative effect of wastewater (Fig. 4). A way of compensating for this effect would be to adjust the achieved P binding capacity with a correction factor. Based on the results of this study, the correction factor would be 0.8 if the experiment is carried out at the low loading rate.

When down-sizing a full-scale filter to laboratory scale, all parameters (such as hydraulic loading rate and influent properties) could be held at the same level while only the residence time would decrease. This approach seems reasonable as the time needed for the reaction of P in the filter material was very short (Fig. 1). However, the reaction time was determined using fresh material only and probably changes over the operational lifetime. Furthermore, the time needed for the reaction does not equal the residence time that is necessary for P retention: the growing of Ca-P crystals that can be retained in the filter might take more time than the actual reaction of dissolved P with Ca. As breakthrough in our experiment occurred earlier than would have been expected from full-scale filter usage, residence time seems to be an important design parameter for both laboratory and full-scale filters, at least for the material investigated in this study. To increase residence time in laboratory filters, however, would considerably prolong the duration of the testing.

## Conclusions

The effects of hydraulic loading rate and influent source on the P binding capacity of *Filtralite^®^P* was investigated in a replicated 2^2^ factorial laboratory experiment. P_tot_ binding capacity was significantly affected by both hydraulic loading rate and influent source. Hydraulic loading rate affected P_tot_ binding capacity more strongly than influent source. An interaction effect of the two factors was not present. Increasing loading rate from 100 to 1200 L m^−2^ d^−1^ caused the P_tot_ binding capacity to decrease from 1.15 to 0.07 g kg^−1^ for wastewater filters and from 1.38 to 0.30 for phosphate solution filters. At a loading rate of 100 L m^−2^ d^−1^, the average P_tot_ binding capacity of wastewater filters was 1.15 g kg^−1^ as opposed to 1.38 g kg^−1^ for phosphate solution filters. Hence, altering these two factors in the laboratory can lead to misleading results that do not reflect a material’s expected performance in field.

The time needed for the reaction of P in fresh filter material was determined to be 5 minutes when phosphate solution was used. Using wastewater, the reaction took longer: after 15 min, the remaining dissolved P in the solution was about 1%. This suggests that a rather short residence time in the filter would be sufficient. However, the reaction times were determined using fresh material and might change with time. Furthermore, as breakthrough in the experiment was reached unexpectedly quickly compared to full-scale filters, there is evidence that it is important to consider not only hydraulic loading rate but also residence time.
